# Establishing human immune system mice: application, opportunity, and challenges

**DOI:** 10.1038/s12276-026-01777-1

**Published:** 2026-07-03

**Authors:** Pei-Chuan Chiang, Hung Lee-Chan, Yungling Leo Lee

**Affiliations:** 1 Institute of Biomedical Sciences, Academia Sinica, Taipei, Taiwan; 2 College of Public Health, China Medical University, Taichung, Taiwan

**Keywords:** Translational immunology, Animal biotechnology

## Abstract

Human immune system (HIS) models offer a promising solution to bridge clinical trial by providing a more functional platform in studying disease mechanisms and evaluating therapeutic responses. This Review provides an overview of various strains of immunodeficient mice used for human immune cell development and their current applications in disease modeling. We highlight the utility of single-cell RNA sequencing in HIS mice as a powerful approach for identifying drug targets and elucidating cellular mechanisms. Furthermore, we discuss four major challenges in HIS model establishment and summarize recent advancements aimed at enhancing human immune cell development. Finally, we present preclinical examples of HIS mice as disease platforms supporting clinical trials, illustrating their role as essential tools for drug development.

## HIS mice revolutionize drug development and disease modeling

Human immune system (HIS) mice have emerged as a pivotal tool in preclinical research, bridging the gap between murine models and human clinical trials. These models, developed by engrafting human hematopoietic stem cells into immunodeficient mice, provide a platform to study human immune responses and drug development. This Review outlines the evolution of HIS mice, highlighting advancements such as the introduction of human cytokines and genetic modifications to improve human cell engraftment and immune cell development. Key findings include enhanced human myeloid and lymphoid cell differentiation, crucial for studying cancer and infectious diseases. These improvements address the limitations of traditional mouse models, offering a more accurate representation of human immune responses. The study underscores the potential of HIS mice in evaluating novel therapies and combination treatments.

This summary was initially drafted using artificial intelligence, then revised and fact-checked by the author.

## Introduction

From the past until now, small animal models, especially mouse model, provided a tool to investigate human immunity. Although human and mouse have similar immune cell types, their immune cell compositions and responses show discrepancies^1^. Therefore, the mice model for preclinical studies often leads to disappointing or unpredictable clinical trial outcomes^2^. Human immune system (HIS) mice are generated by transplanting human hematopoietic stem cell (HSC) or peripheral blood mononuclear cell (PBMC) to establish a functional HIS, providing a valuable model for studying immune responses and drug development^3^.

Traditionally, irradiated immunodeficient neonates received 10^5^–10^6^ of human CD34^+^ HSC or PBMC via intrahepatic or intravenous injection^4^. The engraftment of human CD45^+^ cells in the PBMCs of recipient mice typically reaches 40–60% by 12 weeks post-transplantation. By contrast, CD34^+^ HSC recipient mice were characterized by a higher proportion of B cells (30–70%) and a lower proportion of T cells (10–50% at 12–14 weeks post-transplantation), along with a low percentage of myeloid cells (1–10%)^5,6^. However, the lack of human key factors causes immature human innate cells, and the absence of a human thymus for T cell selection leads to impaired mouse physically bound and presented by specific major histocompatibility complex (MHC)-restricted T cells^7^. Consequently, engineering immunodeficient mice to support the differentiation of human immune cells has become a crucial alternative.

## Advancing HIS development through the engineering of immunodeficient mice

### History of immunodeficient mice for human engraftment

**Fig. 1:**
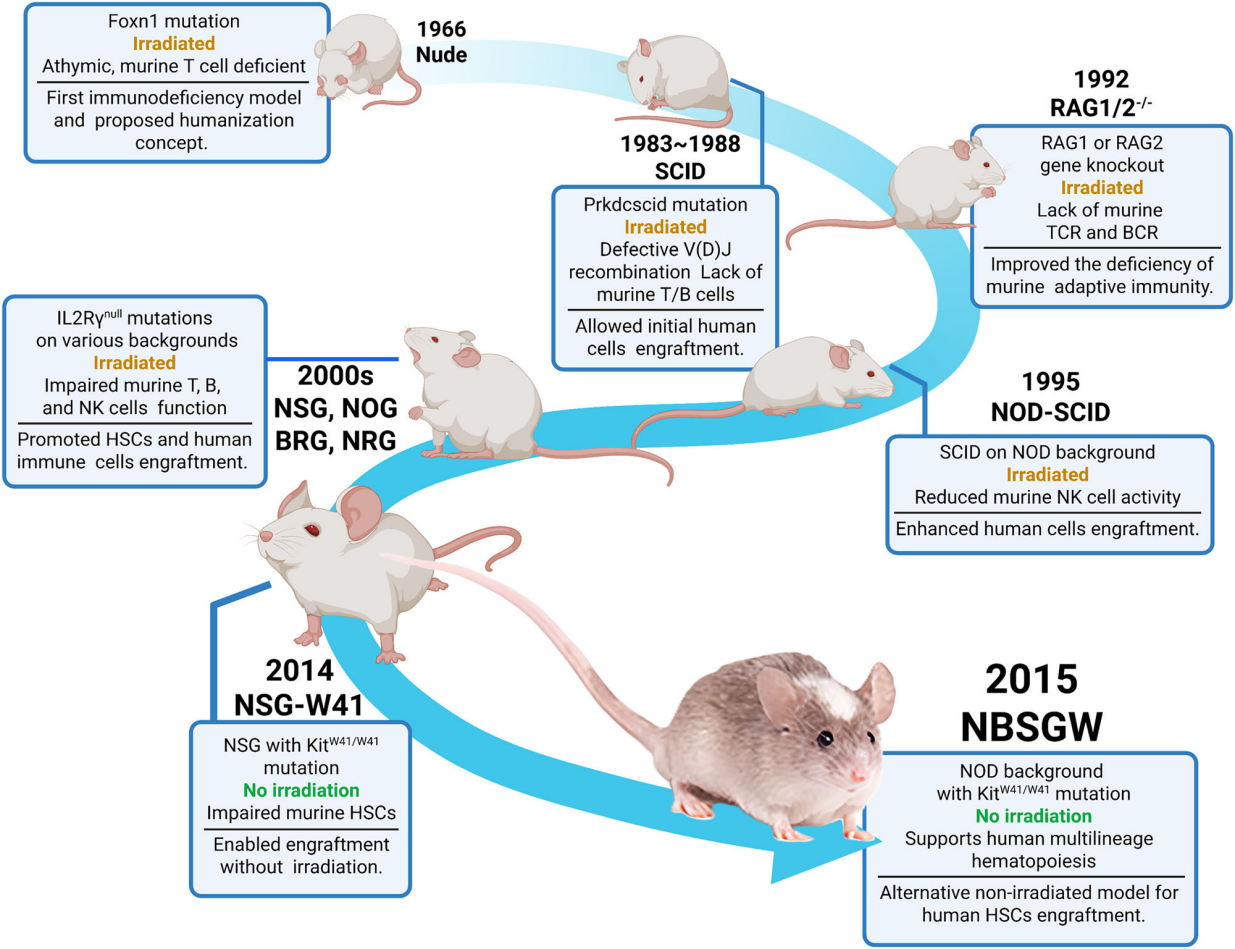
Evolution of immunodeficient mice to enhance human cell engraftment. The evolution of immunodeficient mice for human immune system models is shown. These models have evolved from initially targeting T cell dysfunction to the ablation of the entire adaptive immune system. Since 2014, the strategy of impairing murine HSC differentiation not only improves human cell transplantation efficiency but also increases mouse survival without the need for irradiation. BCR B cell receptor, HSC hematopoietic stem cell, NK natural killer, NOD nonobese diabetic, SCID severe combined immunodeficiency, TCR T cell receptor.

In 1966, Flanagan’s report on the Foxn1 nude mice mutation introduced the idea of humanization for preclinical studies^8^. Seventeen years later, in 1983, the first severely immunocompromised SCID (severe combined immunodeficiency) mice were developed^9–11^. These mice exhibit defective V(D)J recombination owing to a Prkdc gene mutation, leading to a loss of T and B cell function, in which V is variable, D diversity, and J joining. The first HIS mice were described in a 1988 publication, with results demonstrating successful engraftment and maintenance of human lymphoid cell activity in SCID mice for over 5 weeks post-transplantation^12^. To improve the deficiency of mice adaptive immunity for human immune cells transplantation, the RAG1/2^−/−^ mice were generated in 1992, which completely lack functional T and B cells^13,14^. However, engraftment levels remained low until 1995 and 1996, when SCID mice bred onto the non-obese diabetic (NOD) background were developed^15–17^. Later, a mutation was identified in the IL-2 receptor common γ-chain, which mediates signaling for multiple cytokines. This deficiency severely impairs the murine immune system, significantly enhancing human cell engraftment^18^. This discovery led to the development of mice strains such as NOG or NSG (NOD-SCID-IL-2Rγ^null^)^7,19,20^, BRG (BALB-c-RAG2^−/−^IL-2Rγ^null^)^21–23^, and NRG (NOD-RAG1^−/−^IL-2Rγ^null^)^24,25^. In 2009, it was reported that the deficiency of Kit alleles facilitates efficient human HSC engraftment in non-conditioned allogeneic recipient mice. The results indicated that the mutation of Kit alleles allows for efficient human HSC engraftment in recipient mice without the need for irradiation^26^. Building on this concept, subsequent studies have explored strategies to further improve human HSC engraftment in murine models by targeting the KIT signaling axis. Cosgun et al.^27^ were the first to develop the NSG-W41 (NOD.Cg-Kit^W-41J^ Prkdc^scid^ Il-2rg^tm1Wjl/^WaskJ) mice model by introducing the hypomorphic Kit^W41/W41^ mutation into immunodeficient NSG mice. This genetic modification impaired endogenous murine HSC function, thereby opening up bone marrow niches and enabling efficient human HSC engraftment without the need for irradiation. McIntosh et al.^28^ generated NBSGW strain (NOD.Cg-*Kit*^*W-41J*^ *Tyr* + *Prkdc*^*scid*^ *Il-2rg*^tm1Wjl^/ThomJ) by introducing a Kit mutation in a slightly different immunodeficient background. This strain similarly supported long-term, multilineage human hematopoiesis. These mice models provide a promising avenue for preclinical research (Fig.1).

### Specific gene transduction driving human immune cell development

Despite substantial progress in developing immunodeficient mice for human HSC engraftment, current HIS mice still exhibit inherent limitations in fully recapitulating the complexity of the HIS. These limitations primarily arise from the murine microenvironment, which lacks critical human-specific factors required for proper human immune cell development. Consequently, HIS models without further optimization fail to accurately reflect the physiological composition of the HIS in vivo. In the following sections, we focus on three major immune cell lineages that can be selectively enhanced through targeted genetic engineering approaches to improve human immune cell development in HIS models.

## Gene engineering of human HSC development

Although human HSCs can be engrafted into first-generation immunodeficient mice (NSG, NOD, NRG, and BRG strains), their poor engraftment rate and the short survival of these HIS mice have limited their utility in human studies. Human thrombopoietin (TPO) has been reported to support HSC maintenance and differentiation^29,30^. The development of TRG (Rag2^−/−^ Il-2rg^−/−^ 129xBalb/c Thpo^tm1.1(TPO)Flv^), MITRG (TRG-Csf1^tm1(CSF1)Flv^ Csf2/Il-3^tm1.1(CSF2,IL-3)Flv^ Il-2rg^tm1.1Flv^), and MISTRG (MITRG-Tg(SIRPA)1Flv/J) HIS mice in Flavell’s laboratory showed a stable and high engraftment rate of human CD45^+^ cells^31,32^. Similarly, human stem cell factor (SCF, also known as KITLG) has been shown to promote the survival, proliferation, and expansion of HSCs^33^. The expression of SCF in NSG-SGM3 (NOD.Cg-*Prkdc*^*scid*^ *Il-2rg*^*tm1Wjl*^ Tg(CMV-IL-3, CSF2, KITLG)1Eav/MloySzJ) HIS mice also enhanced human myeloid cell development^34^.

Signal-regulatory protein α (SIRPα), a phagocytosis inhibitor receptor, was first characterized in studies published in the 2000s^35^. SIRPα recognizes CD47, which functions as an antiphagocytic signal to discriminate viable cells from dying cells^36^. On the basis of this advantage, the BRG strain (BALB/c-RAG2^−/−^IL-2Rγ^null^Sirpa^NOD^, BRGS) expressed human SIRPα on mouse immune cells, reducing murine macrophage phagocytosis of CD47^+^ human immune cells. This modification improved human immune cells persistence and facilitated human hematopoiesis^37^.

Irradiation-mediated damage to the mouse bone marrow microenvironment can significantly impair the differentiation and long-term engraftment of human HSCs^38,39^. Mutations in the mouse Kit alleles of NSG-W41 and NBSGW mice enable the differentiation of human HSCs without the need for irradiation. This new generation of mice not only preserves the vital bone marrow niche for human HSC development but also improves mouse survival and extends the experimental period for HIS mice models^27,28^.

Over the past few decades, the development of next-generation immunodeficient mice, such as MISTRG, BRGS, and NBSGW, has significantly improved the engraftment of human HSC. These next-generation mice models have established the basis for studying myeloid and lymphoid cell lineages. With mature gene engineering technique, scientists can now modify specific human and mouse genes to focus on a single type of HSC-derived immune cells in HIS mice models. The continuous refinement of new development of HIS will increasingly contribute to clinical studies (Table 1).

**Table 1:** Comparison of next-generation immunodeficient mice for human hematopoietic stem cell engraftment.

Category	Mouse strain	Genetic modifications	Functional improvements	Limitation	Refs.
Irradiation	TRG	Human thrombopoietin (TPO) replaced mouse TPO in Rag2^−/−^ Il-2rg^−/−^ 129xBalb/c (N2) strain	Enhanced human hematopoiesis	Deficient in human thrombopoiesis and erythropoiesis	^31^
	BRGS	Non-obese diabetic (NOD) signal-regulatory protein α (SIRPα) expression in BALB/c-Rag2^−/−^ IL-2Rγ^−/−^	Reduced phagocytosis, enhances human immune cell survival	The affinity of NOD SIRPα for human CD47 is lower than that of human SIRPα	^37^
	MITRG, MISTRG	Human macrophage colony-stimulating factor (M-CSF), IL-3, granulocyte–macrophage CSF, and TPO-expressing Rag2^−/−^ Il-2rg^−/−^ 129xBalb/c (N2) strain	Enhanced human hematopoiesis and myeloid cells development	Human macrophages phagocytose mouse red blood cells, leading to anemia	^32,40^
	NSG-SGM3 (NSGS)	Human stem cell factor (KITLG), IL-3, and GM-CSF (CSF2) encoded NOD-SCID-IL-2Rγ^null^ strain	Enhanced human hematopoiesis, myeloid cells, and mast cells development	Low human hematopoietic stem cell (HSC) maintenance and myeloid cell development	^34^
Non-irradiation	NSG-W41, NBSGW	Introduced the mouse hypomorphic Kit^W41/W41^ G-to-A point mutation into immunodeficient NSG background	Enhanced human hematopoiesis and erythropoiesis by impaired mouse hematopoiesis without irradiation	Human hematopoiesis-induced macrocytic anemia	^27,28^

## Gene engineering of human myeloid lineage

Innate immunity is crucial for cancer research owing to the cytotoxic functions of natural killer (NK) cells and the antigen-presenting capacity of dendritic cells (DCs) and macrophages, which bridge the activation of adaptive immunity. Therefore, establishing myeloid cell dominant HIS mice is critical for elucidating the pivotal role of myeloid cells in shaping effective cancer therapies. Different combinations of IL-3, IL-6, macrophage colony-stimulating factor (M-CSF or CSF1), granulocyte–macrophage CSF (GM-CSF, CSF2), and Fms-related tyrosine kinase 3 ligand (Flt3L) are commonly used to promote myeloid cell differentiation and maturation in HIS mice. The most famous strain for myeloid cell differentiation HIS mice is the MITRG and MISTRG mice, which were developed in Flavell’s laboratory. These mice expressed human cytokines such as M-CSF, IL-3, GM-CSF, and TPO. This expression enhanced the differentiation of myeloid cells, including macrophages, monocytes, and granulocytes. The development of MISTRG HIS mice significantly improved human myeloid cell function, providing valuable preclinical models for studying human innate immunity^32,40^.

Flt3L is a key hematopoietic growth factor that drives the development of various human immune cells, notably cDCs and B cells^41^. Flt3L signals through its receptor Flt3 (CD135), which is present on HSC as well as their subsequent differentiated progenitor cells^42^. Its shares structural similarities with both SCF and M-CSF. It is interesting that the human Ftl3L showed high identity to mouse Flt3L. Owing to their sequence similarity, mouse and human FLT3L can exhibit cross-species activity^43^. Administrating recombinant human Flt3 ligand to Flt3-deficient immunodeficient mice selectively boosted human DC differentiation, creating a more robust immune response platform for studying human antigen-presenting cell biology^44^.

The recognition of aberrant MHC class I (HLA-A) expression by NK cells confers a significant advantage in tumor cell elimination. Unlike T cells, NK cells do not rely on T cell receptor (TCR)-MHC restriction, which allows for their allogeneic use in cancer therapy^45^. Therefore, NK cells serve as the first line of defense against tumorigenesis, exerting both cytotoxic and immunomodulatory effects that suppress cancer progression. Human IL-15 has been reported to promote NK cell development through the JAK–STAT, MAPK–ERK, and PI3K–AKT–mTOR pathways^46^. The insertion of human IL-15 into immunodeficient mice is the most commonly used strategy to establish NK cell-based HIS mice models (Table 2). The SRG-15 strain, for instance, which expresses human SIRPα and IL-15 on NRG (NOD-RAG2^−/−^IL-2Rγ^null^) background, supports robust NK cell development for investigating human NK cell biology and evaluating NK cell-based therapies^47^.

**Table 2:** Comparison of next-generation immunodeficient mice for human myeloid cell development.

Category	Mouse strain	Genetic modifications	Functional improvements	Limitation	Refs.
Monocyte development	NSG-colony-stimulating factor (CSF)1	Introduced human CSF1 gene on the NSG background	Improved the function of human macrophages and monocytes	Homozygous males do not produce viable sperm. Mice are kept hemizygous owing to fertility issues	^156^
	hG-CSF KI	Human granulocyte–macrophage (GM)-CSF knock-in with mouse G-CSF receptor (CSFR) deletion on the NOG background	Enhanced human myeloid cells, especially neutrophil development	High expression of human GM-CSF leading to exhaustion of myeloid progenitors	^157^
	NOG-EXL	Introduced human GM-CSF and IL-3 gene on the NOG background	Promoted the development of human macrophages with long-term stability of human hematopoietic stem cell (HSC)	Low human cytokine expression results in lower myeloid cells development and maturation	^158^
	NSG-SGM3 (NSGS)	Human stem cell factor (SCF) (KITLG) IL-3 and GM-CSF (CSF2) encoded NOD-SCID-IL-2Rγ^null^ strain	Enhanced human hematopoiesis, myeloid cells and mast cells development	Low human HSC maintenance and myeloid cell development	^34^
	NSG-Quad	Expressed human GM-CSF, IL-3, SCF and M-CSF gene on the NSG background	Enhanced human monocyte and tissue-resident macrophage development	Overexpression of hM-CSF induced monocyte hyperactivation syndrome	^159^
	hIL-6 NOG	Expressed human IL-6 on NOG background	Enhanced myeloid development after HSC engraftment enables studies of tumor-infiltrating macrophages	The study was restricted to monocytes. The myeloid cells were immature	^160^
	MITRG	Human M-CSF, IL-3, GM-CSF, and TPO-expressing Rag2^−/−^ Il-2rg^−/−^ 129xBalb/c (N2) strain	Promoted macrophage, monocyte, and granulocyte differentiation	Human macrophages phagocytose mouse red blood cells, leading to anemia	^32^
	MISTRG	Human signal-regulatory protein α transgenic MITRG mice	Improved innate immune functionality	Human macrophages phagocytose mouse red blood cells, leading to anemia	^32,40^
	MITRGGGR	Human GM-CSF knock-in with mouse G-CSFR deletion in the MISTRG strain	Enhanced human hematopoiesis and myeloid cells, especially neutrophil development	Multiple gene modification results in poor breeding efficiency	^161^
Dendritic cell (DC) development	NOJ-Fms-related tyrosine kinase 3 ligand (Flt3L)/GM-CSF	Human Flt3L and GM-CSF plasmids were introduced into the NOJ background via IVT	Enhanced human myeloid cells, especially human conventional DCs (cDCs) development	This mouse is more susceptible to environmental factors than standard immunodeficient mice	^162^
	NSG Flt3KO hFLT3LG Tg (NSG-FLT3)	Human Flt3L knock-in in mouse Flt3-deficient NSG mice	Enhanced survival, proliferation, and maturation of human cDCs	This mouse is more susceptible to environmental factors than standard immunodeficient mice	^163^
Natural killer (NK) cell development	SRG-15	Sprague–Dawley Rag2^−/−^ IL-2Rγ^−/−^ mice expressing human IL-15	Improved human NK cells development	The mice are maintained as hemizygous due to fertility issues	^47^
	hIL-15 NOG	NOG mouse expressing human IL-15 cytokine	Predominant differentiation of human NK cells following human HSC engraftment compared with the base NOG mouse	High IL-15 may cause NK exhaustion, decreasing CTL activity and mouse survival	^164^
	NSG-Tg (Hu-IL-15)	NSG mouse expressing human IL-15 cytokine	Human IL-15 expression promotes human NK cell development in humanized immunodeficient mice	The mice are maintained as hemizygous due to fertility issues	^165^
	NSG-SGM3-IL-15	Expressed human IL-15 on NSG-SGM3 mice	Improved human NK cell development in myeloid cell-dominated immunity	This mouse is more susceptible to environmental factors than standard immunodeficient mice	^166^
	NSG-FLT3-IL-15	Transgenic expression of human IL-15 and Flt3L in Flt3-deficient NSG mice	Promoted human NK cell development in diverse human hematopoiesis	This mouse is more susceptible to environmental factors than standard immunodeficient mice	^167^

## Gene engineering of human lymphoid lineage

Adaptive immunity is the primary defense against all of the antigen-dependent diseases. However, the absence of human cytokines and HLA-A expression in most immunodeficient mice results in dysfunction of human HSC-derived T cells in HIS mice. To enhance T cell development, Coppin et al.^48^ introduced human IL-7 on NBSGW background mice (NBSGW-IL-7). This model supports efficient T cell differentiation in the thymus and sustained peripheral T cell expansion following transplantation with human HSC, without inducing abnormal lymphoproliferation. By contrast, the development of BRGS A2DR2 (BRGS-Tg (HLA-A*02-HHD, HLA-DR2)/Rj) and DRAGA mice (NOD-HLA-A2.HLA-DR4 RAG1^−/−^IL-2Rγ^null^) expressed both HLA class I and II molecules on the immunodeficient mice. This model allows for improved development of functional human T and B lymphocytes, including antigen-specific responses, cytokine production, and immunoglobulin class-switching. These models offer distinct advantages for studying HLA-matched HISs in antigen-dependent immunization studies^49,50^ (Table 3).

**Table 3:** Comparison of next-generation immunodeficient mice for human T cell development.

Category	Mouse strain	Genetic modifications	Functional improvements	Limitation	Ref.
Maturation	NBSGW-IL-7	Human IL-7-encoded NBSGW strain	Improved human T cell development	Human hematopoiesis-induced macrocytic anemia	^48^
Thymopoiesis	NSG-HLA-A2/HHD	Expressed a human B2M protein covalently linked to the human HLA-A2 heavy and light chains on the NSG background	Supported human CD8 T cell education and maturation	Lack of human HLA-DR expression. Only HLA-matched hematopoietic stem cell (HSC) can be used for engraftment	^168^
	NSG DR4	The DR4β (NT) transgene was modified to mirror endogenous mouse major histocompatibility complex (MHC) class II on the NSG background	Supported human CD4 T cell and B cell development for autoimmune diseases model	Only HLA-matched HSC can be used for engraftment	^169^
	BRGS A2DR2	Expressed human HLA-A2 and HLA-DR2 gene on the BRGS background	Supported the thymopoiesis of HLA-A2 and DR2 T cells to generate functional human T cells	Lower binding affinity of signal-regulatory protein α (SIRPα). B cells were immature. Only HLA-matched HSC can be used for engraftment	^50^
	DRAGA	Rag2^−/−^ IL-2Rγ^−/−^ NOD mice expressing human HLA-A2.1 and HLA-DRA/HLA-DRB1*0401	Enhanced HLA-matched HSC engraftment and subsequent human T cell and B cell development	Only HLA-matched HSC can be used for engraftment. The mice are difficult to breed	^49^

The interaction between innate and adaptive immunity is crucial for investigating the full spectrum of immune responses in humans. To achieve multilineage differentiation of human lymphoid and myeloid cells, Yu et al.^51,52^ and Richard A. Flavell’s laboratory introduced human IL-6 into mouse Flt3-deficient NSG (NSGF6) and MISTRG (MISTRG6) HIS mice. The establishment of these HIS mice supports the long-term engraftment of HSC and the development of diverse human immune cells.

Lymph nodes have a crucial role in the maturation and activation of innate and adaptive immune cells. However, in most immunodeficient mice, the absence of the IL-2 receptor gamma (IL-2rg) gene eliminates IL-7 signaling. This signaling impairment results in the loss of lymphoid tissue inducer cells, which are essential for the development of secondary and tertiary lymphoid tissues. To overcome this deficiency, the BRGST strain was generated by incorporating thymic stromal lymphopoietin (TSLP) into the BRGS background. This mouse not only improved myeloid cell development but also enhanced germinal center formation, class-switched immunoglobulin production, somatic hypermutation, and neutralizing antibody production post-immunization^53^. Furthermore, the expression of TSLP in mice promoted both myeloid and lymphoid immune responses, particularly enhancing T cell differentiation and T_H_2-type immunity (Table 4).

**Table 4:** Comparison of next-generation immunodeficient mice for human lymphoid organ development.

Category	Mouse strain	Genetic modifications	Functional improvements	Limitation	Ref.
IL-6- mediated	NSGF6	Human IL-6 knock-in in mouse Flt3-deficient NSG mice	Supported human hematopoietic stem cell differentiation with better engraftment. Multilineage differentiation of human lymphoid and myeloid cells was observed	Female mice showed better engraftment results to male mice	^51^
	MISTRG6	MISTRG strain with a transgene for human IL-6	Supported human myeloid cells development and improved B cells responses	Human macrophages phagocytose mouse red blood cells, leading to anemia	^52^
Thymic stromal lymphopoietin (TSLP)-mediated	NSGFT	Human TSLP knock-in on mouse Flt3-deficient NSG mice	Promoted human hematopoiesis. Expressed TSLP enhanced lymphoid organ reconstitution	Female mice showed better support human hematopoietic stem and progenitor cell engraftment than males	JacksonLab (039497)
	BRGST	BRGS strain with a transgene for TSLP	Improved germinal center formation, antibody class-switching, T_H_2 differentiation, and adaptive responses	Lower binding affinity of signal-regulatory protein α. Lack of intestinal Peyer's patches	^53^

## HIS mice model for preclinical research and drug development

### HIS infection disease model

Most infectious diseases exhibit species specificity, which limits the applicability of traditional mice models in studying human pathogens. This species barrier often leads to significant discrepancies between murine and human immune responses, thereby impeding accurate investigations of disease mechanisms and therapeutic interventions. To address these limitations, several alternative models have been developed, including HIS mice, organoids, and in vitro culture systems. Among these, HIS mice models have become indispensable tools for studying human-specific pathogens, particularly those with strict species tropism. These models have enabled researchers to investigate viral replication, pathogenesis, human immune responses, and treatment effects for preclinical research.

#### *Salmonella*

HIS mice have been used to study *Salmonella enterica* subsp. *enterica*serovar Typhimurium (S*.*Typhi), a pathogen that causes systemic disease in humans but fails to induce progressive infection in standard murine hosts. CD34^+^ HSC HIS mice infected intravenously or intraperitoneally with S*.*Typhi develop granulomatous inflammation with mononuclear infiltration in the spleen, mimicking human typhoid symptoms^54,55^. Virulence determinants from S.Typhi were identified using transposon mutant library screening in HIS mice models. Several key genes, including those in *Salmonella* pathogenicity islands (SPI-1, SPI-2, and SPI-6) as well as *rseP* and mgtC, have important roles in S. Typhi survival and disease progression in vivo^56^.

#### Varicella-zoster virus

HIS mice models have also been used to study human-tropic viruses that exhibit strong tissue specificity, such as varicella-zoster virus (VZV). Traditional mice models fail to support productive VZV infection owing to the strict species restriction of the virus. Nevertheless, in human PBMC (huPBMC) HIS mice with human skin xenografts, VZV replicated in both the graft and human T cells. This viral replication led to vesicular skin lesions resembling those observed in patients with varicella^57^. This model provided one of the earliest examples of how HIS mice could overcome interspecies barriers to study human viral pathogenesis in vivo.

#### Hepatitis virus

Human liver chimeric mice, such as uPA/SCID (SCID-uPA^+/+^) and FRG (Fah^−/−^Rag2^−/−^IL-2Rγ^null^) models transplanted with human hepatocytes, have been widely used to study hepatotropic viruses including hepatitis B virus and hepatitis C virus. These HIS mice models enable the in vivo investigation of host–pathogen interactions, antiviral immunity, and liver-specific injury mechanisms^58^. This humanization strategy offers a platform to evaluate therapeutic interventions and dissect immune-mediated pathogenesis within a human-like liver environment.

#### Respiratory diseases

HIS mice models for influenza infection offer a human-like immune environment for preclinical test. The human lung tissue-xenografted HIS mice model provides a robust platform for analyzing lung immune cell infiltration, tissue-resident immunity, and the systemic production of virus-specific neutralizing antibodies. RNA-sequencing (RNA-seq) analysis of human lung tissue xenografts performed by Wang et al. revealed the expression of interferon-stimulated genes (for example, IFI44L, OAS2, ISG15, and IFITM1) and chemokine genes (for example, CXCL17, CXCL6, and CXCL1), which mimic human lung microenvironment responses under viral infection conditions. This preclinical platform provides a comprehensive analysis for influenza vaccine development^59–62^.

COVID-19 models have been successfully established in HIS mice by the transplantation of human lung tissue or the overexpression of human ACE2 (hACE2) in the lungs. This platform recapitulates key features of human COVID-19 symptoms, such as alveolar damage, immune cell infiltration, and the activation of human T cells and cytokine production^63^. To investigate the specific immune cell mechanism involved in vaccine treatment against viral infection, two mice strains were reported to study SARS-CoV-2 infection in HIS mice models.

The MISTRG-encoded human IL-6 MISTRG6 mice model with hACE2 expression by adeno-associated virus serotype 9 (AAV9) in lung of mice allows susceptibility to SARS-CoV-2 infection by supporting the development of human innate immune cells. MISTRG6-infected mice demonstrated chronic inflammation primarily due to the hyperactivation of macrophages, DCs, and neutrophils. The prolonged inflammatory responses in these models make them a crucial platform for studying immune dysregulation and testing therapies against chronic inflammation and viral persistence^64^.

Engrafted with hACE2-expressing epithelial and endothelial cells, DRAGA mice support SARS-CoV-2 infection and exhibit human-specific immune responses, notably T cell-mediated immunity. DRAGA mice are particularly useful for studying antigen presentation and T cell interactions with viral antigens during chronic infection. Similar to MISTRG6, DRAGA mice develop persistent infection and sustained immune activation, making them suitable for investigating the long-term immunological consequences of COVID-19 and for evaluating immunotherapeutic interventions^65^.

Both models provide valuable insights into the pathophysiology of chronic COVID-19. They support the examination of immune responses that closely resemble those observed in humans, offering a deeper understanding of viral persistence and chronic inflammation. Additionally, these HIS mice serve as tools for preclinical testing of therapeutic approaches to explore potential treatments for chronic COVID-19.

### HIS cancer model

Although traditional mice models have contributed to our understanding of cancer biology, cross-species differences still present a significant gap in translating basic research findings to clinical trials. HIS mice tumor models provide a critical platform for investigating the exact human immune responses in tumor microenvironment (TME), which are essential for drug development and efficacy evaluation^66^.

#### Melanoma

Melanoma, an aggressive cancer with a high propensity for metastasis, frequently exhibits resistance to BRAF inhibitors in patients with BRAF-mutated disease. Single-cell transcriptomics of melanoma samples of patients reveal a profoundly immunosuppressive TME. This environment is defined by exhausted CD8⁺ T cells expressing PD-1, TIM-3, and LAG-3, coupled with tumor-associated macrophages (TAMs) whose transcriptional states actively impede antitumor immunity. These immunosuppressive myeloid populations are recognized as central drivers of immune checkpoint blockade resistance in melanoma. The implantation of A375 cells into MISTRG HIS mice enables the study of metastatic melanoma. Through this cell-line-derived xenograft model, human TME-associated mononuclear phagocyte system (MPS) cells have been observed driving tumor progression and metastasis. Single-cell RNA-sequencing (scRNA-seq) further identified MPS4, a distinct myeloid subpopulation characterized by glycolytic reprogramming and chemokine overexpression. This population diverges from canonical M1/M2 polarization and remains transcriptionally conserved in patient samples, providing a mechanistic link between macrophage heterogeneity and melanoma pathogenesis^67^.

#### Triple-negative breast cancer

Triple-negative breast cancer (TNBC) is an aggressive subtype cancer cell, which lacks estrogen receptor (ER), progesterone receptor, and HER2 expression and disproportionately affects younger women. From scRNA-seq, profiling of samples of patients with TNBC has demonstrated a TME dominated by immunosuppressive populations, including M2-polarized TAMs, myeloid-derived suppressor cells (MDSCs), and regulatory T (T_reg_) cells. These cells collectively impair CD8⁺ cytotoxic T cell function, fostering a “cold” tumor phenotype that is largely refractory to PD-1/PD-L1 blockade monotherapy^68^. In particular, elevated MDSC infiltration has been identified as a key predictor of non-response to treatment in patients with TNBC^69^. This immunosuppressive landscape underscores the necessity of combination strategies that simultaneously modulate immune cell recruitment and tumor killing. Combining oncolytic virotherapy with chemokine-mediated immune recruitment represents a promising strategy to overcome the immunosuppressive TME characteristic of TNBC. Ang et al. engineered the chimeric oncolytic adenovirus Ad5F11bSP-Rantes, featuring tumor-selective replication driven by the survivin promoter. Additionally, the virus expresses the chemokine RANTES (CCL5) to recruit NK cells and T lymphocytes into the TME. In a huPBMC HIS mice model of MDA-MB-231 TNBC, intratumoral administration of Ad5F11bSP-Rantes not only achieved an 88.33% tumor inhibition rate but also elevated circulating RANTES levels, enhancing CD3^+^ T lymphocyte infiltration. These results demonstrate the potential of combining oncolytic virotherapy with chemokine-based immune modulation to convert an immunosuppressive TME into antitumor immune responses^70^.

#### Lung cancer

scRNA-seq of samples of patients with non-small-cell lung cancer (NSCLC) reveals a highly immunosuppressive TME. This environment is characterized by exhausted CD8⁺ T cells expressing PD-1, TIM-3, and TIGIT, alongside significant MDSC infiltration that suppresses antitumor immunity via arginine depletion and reactive oxygen species production^71^. These mechanisms drive primary and acquired resistance in more than 70% of patients with NSCLC treated with immune checkpoint inhibitors (ICIs)^72^. Synergistic blockade of multiple immunosuppressive axes is critical to overcome this milieu^73^. On the basis of A549-xenografted HIS mice model, Thakkar et al.^74^ treated the HIS mice with the HMBD-002 antibody to inhibit VISTA (V-domain immunoglobulin suppressor of T cell activation)-mediated immune suppression in myeloid cells. This treatment promoted a T_H_1/T_H_17 antitumor inflammatory response to suppress tumor growth. In addition, Borchmann et al.^75^ used an NSCLC PDX-HIS mouse model to test a tripartite antigen-agnostic combination immunotherapy (TRI-IT). Their findings demonstrated that TRI-IT effectively eradicated poorly immunogenic tumors, offering a strategic model to overcome multilayered immune resistance in NSCLC.

Neuropilin-1 (Nrp-1) is a novel immune checkpoint of interest in NSCLC, known for its role in regulating CD8^+^ T cell memory and long-term antitumor immunity^76^. Despite its potential, its specific function in NSCLC was previously unclear. Zhang et al.^77^ developed a fully human anti-Nrp-1 IgG1 antibody (53-IgG1) via a patient-derived scFv phage library. The efficacy of the antibody was then validated using an A549-xenografted HIS mice model. Treatment with 53-IgG1 significantly reduced tumor volume and weight compared with the IgG1 control group, with no notable adverse effects. Flow cytometric analysis of tumor-infiltrating leukocytes (TILs) revealed markedly elevated CD8⁺ T cell and CD3⁺CD45⁺ cell counts in tumor tissues, suggesting that the antitumor effect was mediated through enhanced CD8⁺ effector T cell infiltration into the TME. Supporting the in vivo findings, in vitro results indicated that 53-IgG1 partially restored the activity of exhausted CD8⁺ T cells from patients with lung adenocarcinoma and increased the proportion of naive and central memory T cell subsets.

#### Colorectal cancer

HIS mice can also be used in colorectal cancer (CRC) model for preclinical evaluation. scRNA-seq profiling of samples of patients with CRC has revealed that microsatellite-stable (MSS) CRC cases exhibit a “cold tumor” phenotype, characterized by high infiltration of immunosuppressive cells. Further analysis of patients with CRC showed that IL-1β-driven MDSC infiltration was a key factor suppressing CD8⁺ T cell function and conferring resistance to anti-PD-1 therapy^78^. These omic-derived insights highlight that converting an immunosuppressive MSS TME to an immune-responsive state necessitates TME-remodeling strategies beyond checkpoint inhibition alone. Addressing the immunosuppressive barriers of MSS tumors, Lang et al.^79^ established MSS-CRC PDX model in HIS mice to evaluate combination therapies. Their findings demonstrated that cabozantinib (a multityrosine kinase inhibitor) synergizes with nivolumab to significantly increase the infiltration of cytotoxic CD8^+^ T cells and granzyme B^+^, TNFα^+^, and IFNγ⁺ leukocytes. This study underscores the utility of HIS mice models in the rational preclinical design of strategies to overcome myeloid-driven immune resistance.

By contrast, microsatellite instability-high CRC subtypes remain clinically responsive to ICI treatment^80^. Using HIS mice bearing HCT116 and SW480 xenografts, Shang et al.^81^ demonstrated the potent antitumor efficacy of BMS202. This small-molecule PD-1/PD-L1 inhibitor significantly suppressed tumor growth, highlighting its therapeutic potential in CRC HIS mice models.

#### Hepatocellular carcinoma

Hepatocellular carcinoma (HCC) originates in hepatocytes and is typically associated with chronic liver disease, especially cirrhosis resulting from chronic hepatitis B or C infection. The liver’s inherently immunotolerant microenvironment substantially complicates HCC immunotherapy. Multi-omic profiling of samples of patients with HCC demonstrated an “immune-exhausted” HCC characterized by terminally differentiated MMP9⁺ TAMs and TREM2⁺ macrophages as key drivers of CD8⁺ T cell exhaustion^82^. Recent insights have identified that growth factor-mediated signaling pathways are pivotal in shaping this immunosuppressive TME. Among these, fibroblast growth factor (FGF) signaling has been known not only for its canonical oncogenic roles in hepatocarcinogenesis but also for its ability to modulate myeloid cell behavior, including recruitment, polarization, and pro-tumoral activity^83^. Tai et al.^84^ demonstrated that combining infigratinib and FGF401 effectively suppresses tumor growth in HCC PDX HIS mice models expressing high levels of FGFR2/3 or FGF19/FGFR4. This combination enhances antitumor immunity by disrupting the FGF/FGFR oncogenic axis, thereby addressing the critical need to reverse myeloid-mediated immunosuppression in HCC.

#### Renal cell carcinoma

Clear cell renal cell carcinoma (ccRCC) represents more than 75% of all kidney cancer cases. This malignancy is characterized by a complex and immunosuppressive TME. Clinical observations and scRNA-seq analyses confirm the presence of four TAM subpopulations with exhausted CD8^+^ T cells and T_reg_ cells. These cells express multiple exhaustion markers such as PD-1 in T cells and CTLA-4 in T_reg_ cells. Furthermore, the HLA-G–LILRB1/2 axis between tumor cells and TAMs is observed to facilitate M2-like polarization. These factors together create a robust barrier that protects the tumor from host immunity and medical intervention^85^. Unfortunately, traditional mice models of renal cancer poorly represent the HIS. This is because certain B7 immune checkpoint members such as HHLA2 are highly expressed in human kidney cancer but have no mouse ortholog^86^.

To address this gap, Wang et al.^86^ engineered immune-restoring chimeric antigen receptor (CAR)-T cells targeting carbonic anhydrase IX (CAIX) in a ccRCC orthotopic HIS mice model engrafted with partially HLA-matched CD34⁺ HSC (hccRCC-NSG-SGM3). This model successfully recapitulates the same pattern of increased exhausted T cells and M2-like macrophages observed in advanced human ccRCC. The profiling of TILs from treated tumors revealed that CAR-T cell therapy enhances tumoricidal cytotoxicity, improves resistance to T cell exhaustion, and promotes T follicular helper–B cell crosstalk. This study specifically highlights how combining CAR-T therapy with immune checkpoint blockade overcomes immunosuppressive barriers.

In summary, HIS tumor models represent a unique and underappreciated platform for mechanistic studies of immunotherapy resistance. Integrating spatial transcriptomics with these models enables high-resolution mapping of resistance-associated immune niches. This approach directly informs the rational design of combination therapies at the preclinical stage.

## HSC-derived human immune cell identified by scRNA-seq

### Characterization of human immune cell subset for therapeutic intervention

HIS mice models integrated with scRNA-seq have become indispensable tools for dissecting human immune cell development and function. Iwabuchi et al.^87^ used HIS mice to identify a previously unrecognized subset of human CD14⁺ DCs with a hybrid transcriptional profile between monocytes and conventional DCs (cDCs). These unique CD14⁺ DCs demonstrated elevated expression of inflammatory genes and antigen presentation machinery, suggesting a crucial role as inflammatory antigen-presenting cells. Moreover, this study identified cDC1 and cDC2 in HIS mice and defined a distinct third population, termed DC3, which exhibited a unique transcriptional signature associated with inflammatory cytokine production. These findings demonstrate the utility of HIS mice for establishing in vivo models of human immune subset differentiation and their potential for multi-omics and functional studies.

Evren et al.^88^ demonstrated that circulating human monocytes in HIS mice differentiate into alveolar-like and interstitial macrophages, effectively recapitulating the cellular heterogeneity of the human lung. Their findings highlight a lineage-specific migration pattern. Specifically, classical CD14⁺ blood monocytes were found to migrate into lung tissue and differentiate into both interstitial and alveolar macrophages. By contrast, non-classical CD16⁺ blood monocytes preferentially gave rise to pulmonary intravascular macrophages. Complementing these fate-mapping approaches, scRNA-seq of more than 28,000 human myeloid cells identified intermediate transcriptional states along the monocyte-to-macrophage differentiation trajectory. These findings illustrate how cellular origin shapes macrophage identity, localization, and functional heterogeneity in vivo. Together, these studies underscore the complexity of human myeloid cell differentiation and the limitations of transcriptomic profiling alone in fully resolving immune cell identity.

In this context, the observed heterogeneity in DC3 populations and the diverse differentiation states of monocytes highlight the need for integrative multi-omics approaches. Integrating CITE-seq with single-cell multiome (ATAC and gene expression) offers a more comprehensive characterization of immune cell subsets^89,90^. CITE-seq captures surface protein expression alongside the transcriptome, whereas single-cell multiome links chromatin accessibility with gene expression at single-cell resolution. Furthermore, candidate regulators identified through such multidimensional profiling can be functionally validated using in vitro perturbation systems, such as gene knockdown or CRISPR-based approaches. Furthermore, CRISPR activation (CRISPRa) and CRISPR interference (CRISCRi) enable precise gene activation and repression, whereas Cas13-mediated multiplex RNA silencing offers additional flexibility. These approaches manipulate gene expression without inducing DNA double-stranded breaks and have been successfully applied in recent perturb-seq studies to dissect regulatory networks^91,92^. Together, the synergy between HIS mice models and multi-omics approaches provides a robust framework for identifying therapeutic targets within the human immune landscape, thereby accelerating the development of next-generation immunotherapy.

### Analysis of human immune cell responses to cancer immunotherapy

Cancer immunotherapy has transformed the treatment landscape for multiple malignancies; however, the complexity of the TME presents significant challenges in predicting and improving therapeutic outcomes. The integration of HIS mice models and scRNA-seq provides a high-resolution platform to dissect immune cell dynamics in the TME, offering mechanistic insights into immunotherapy-driven responses. Luo et al.^93^ used CD34^+^ HSC HIS mice and scRNA-seq to investigate the impact of the BCL-2 inhibitor APG-2575 on the tumor immune microenvironment. Their analysis revealed that APG-2575 reprogrammed immunosuppressive M2-like TAMs toward a pro-inflammatory M1-like phenotype through NLRP3 activation. This phenotypic switch was associated with enhanced CD8⁺ T cell infiltration and an improved response to anti-PD-1 therapy, illustrating the value of HIS mice models in evaluating Immune-based therapies targeting the HIS.

Similarly, Liu et al.^94^ explored the immune landscape in HIS mice treated with dual PD-1 and TIGIT blockade, using scRNA-seq to dissect immune cell populations within the TME. Their study demonstrated that the combination therapy promoted the expansion of CD56⁺ NK cells and CD8⁺ effector T cells while also inducing changes in the myeloid cell compartment, reflecting the complex interplay of immune checkpoint regulation and therapeutic efficacy.

### Investigation of human lymphoid cell diversification and clonal expansion

Adaptive immunity is characterized by the diversification and clonal expansion of lymphocytes following antigenic challenge. Elucidating the development, somatic hypermutation, and antigen-specific responses of human T and B cell repertoires is essential for advancing vaccine design and understanding infectious disease pathogenesis. Combining HIS mice models with scRNA-seq and V(D)J repertoire analysis provides an opportunity to track immune dynamics at clonal resolution in a human-based in vivo environment. Chupp et al.^95^ focussed on adaptive immunity, using the NBSGW-based TruHuX (THX) HIS mice model and scRNA-seq to analyze T and B cell clonal diversity following immunization. By incorporating V(D)J sequencing analysis, they demonstrated the development of a diverse and polyclonal TCR and B cell receptor repertoire. Their study revealed the formation of germinal centers and the presence of somatically hypermutated, class-switched immunoglobulin genes, indicating active affinity maturation. In response to both vaccine and SARS-CoV-2 pseudovirus infection, expanded B cell clones were identified alongside antigen-specific transcriptional signatures, reflecting functional engagement of adaptive immune responses. These findings establish the NBSGW THX mice model as a valuable platform for studying antigen-specific immune responses and for evaluating vaccine-induced immunity in vivo.

In summary, the aforementioned studies showed the comprehensive analysis of single-cell transcriptomics and HIS mice models in providing high-resolution insights into human immune cell differentiation, function, and therapeutic modulation in vivo. This integrated approach offers a significant opportunity to advance our fundamental understanding of human immunity and accelerating progress in preclinical research for both infectious diseases and cancer immunotherapy.

## Improving human immune system development for clinical use

### Survival of recipient mice

Excluding deaths from graft-versus-host disease or irradiation, most HIS mice have a shorter lifespan. This is primarily due to impaired murine red blood cell (RBC) development, which can lead to hypoxia or death. Despite the functional limitations of human RBCs in mice, clodronate liposomes (CloLip) treatment can enhance mouse survival. This is achieved by depleting mouse macrophages to prevent the phagocytosis of human RBCs^96,97^. Yamaguchi et al.^98^ showed that the murine complement system can also reduce human RBC survival. Knockout of complement C3, in conjunction with CloLip treatment, has been shown to restore human RBC levels and prolong mice survival.

### Inbreeding issues

Maintaining the genetic stability of mice strains is essential for ensuring a consistent supply of reliable models^99^. In vitro fertilization offers a valuable method for preserving fertilized eggs and ensuring a stable production of HIS mice^100^. However, extensive gene knockouts or deletion-induced loss of function can result in immunocompromised mice having abnormal organ development, hematopoietic dysfunction, and infertility^101^. For example, β2-microglobulin-deficient mice, which lack MHC class I expression, exhibited reduced litter sizes and decreased mating efficiency^102^. Another study found out that the knockout of the mouse Flt3 receptor in mice may disrupt embryonic development through PI3K pathway^103^. Therefore, strategies to minimize inbreeding and refine genetic modifications are crucial for improving mice fertilization.

### Human adaptive immunity

#### T cell maturation and differentiation

T cell immune responses are critical readouts in oncology research and vaccine development. Through positive and negative selection, this diverse TCR repertoire enables T cells to respond to a wide range of diseases by secreting cytokines and cytotoxic clearance. However, the absence of human HLA in CD34⁺ HSC HIS mice leads to impaired human T cell development. This challenge is further underscored by humanized systemic lupus erythematosus (SLE) models, in which a HLA mismatch contributes to incomplete T and B cell functional reconstitution^104^. Consequently, introducing human HLA expression in these mice is necessary to enable proper T and B cell functional reconstitution and to support mechanistic studies of adaptive immune diseases.

Transplantation of human thymus tissue represents the optimal method for generating a complete T cell repertoire in HIS mice, but the ethical considerations impede their use in translational studies. Recently, iPSC-derived artificial thymic organoids offer a promising way to generate HIS mice with a functional human thymus^105^. This methodology uses a four-stage differentiation process to produce induced pluripotent stem cell (iPSC)-derived thymic epithelial cells (iPSC-TECs), which are then combined with human CD34^+^ HSC on decellularized murine scaffolds to form HPC/iPSC-TEC organoids. Transplantation of these organoids into NSG mice has enabled human T and B cell development. This approach holds significant implications for the study of human immunity and the development of human disease models.

An alternative approach to promote T cell immunity would involve the transfection of human HLA genes into the mouse thymus. The combination transfection of HLA-A2 and cytokines by AAV showed the comprehensive innate and adaptive cellular responses for cancer nano-vaccine development^106,107^. Generating monochain HLA class 1 transgenic mice showed antigen-specific cytotoxic responses^108^. In addition, the development of DRAGA and BRGS-A2DR2 transgenic mice, which expressed both HLA class I and class II genes, showed significant T cell responses in an immunization model^50,109^.

The differentiation of effector T cells from naive CD4⁺ T cells depends on cytokine signals^110^. However, many cytokines are species-specific owing to evolutionary divergence between humans and mice^111^. Among CD4⁺ T cell subsets, T_reg_cells are essential for regulate autoimmune disease and cancer. The lack of human cytokines results in impaired T_reg_ function and leads to the overactivation of autoreactive T and B cells within SLE HIS mice models^104^. Although mouse transforming growth factor-β exhibits substantial cross-reactivity in supporting human T_reg_ development, mouse IL-2 has limited affinity for human IL-2 receptors and is insufficient to drive human T_reg_ differentiation in HIS mice^112–114^. This has been demonstrated by AAV-mediated overexpression of IL-2, which promotes T_reg_ expansion in HIS mice^115^. Furthermore, low-dose IL-2 treatment expands T_reg_ cells and alleviates disease severity in the 2,4,6-trinitrobenzenesulfonic acid-induced colitis model in HIS mice^116^. Thus, expression of human IL-2 is required for efficient generation and functional study of T_reg_ cells in HIS mice. Interestingly, expression of human SCF, GM-CSF, and IL-3 in NSG-SGM3 HIS mice not only enhances myeloid cell development but also promotes peripheral expansion of T_reg_ cells. This observation suggests that myeloid-derived signals support T_reg_homeostasis and reinforce their suppressive capacity against polyclonally activated T cells^117^. Building on this model, Sitta et al.^118^ established MDA-MB-231 tumor-bearing NSG-SGM3 HIS mice to evaluate immuno-oncology strategies in TNBC. Antitumor efficacy of microbubble-protected oncolytic virotherapy (MB/OV) was assessed either alone or in combination with pembrolizumab. Immunohistochemical analyses of tumor-infiltrating lymphocytes showed that MB/OV treatment increased CD8⁺ cytotoxic T cell infiltration while reducing T_reg_ abundance within the tumor. These findings highlight the utility of this HIS mice model for functionally elucidating functional T_reg_ responses to novel therapies. Together, human cytokines are critical for supporting T_reg_ differentiation and function in HIS mice, improving their relevance for studying human immune regulation.

#### Immunoglobulin production

Humoral response is essential for human therapeutic antibody development and preclinical vaccine evaluation. Technologies such as hybridoma, genomic engineering, and interspecies immunization are used for antibody production. Human immunoglobulin repertoire mice offer the advantage of producing human-like antibodies by incorporating human V(D)J gene segments from the human antibody repertoire into the mouse genome^119,120^. The antigen-specific antibody genes isolated from human immunoglobulin repertoire mice can be cloned for large-scale antibody production and engineered for isotype switching via molecular techniques for therapeutic applications^121^. However, the gene size and complexity of the human Ig loci present significant challenges to stable genomic integration, leading to gene recombination instability and class-switching issues^122^. Therefore, a natural human B cell from HIS mice provides a potential solution to these challenges.

HIS mice have been shown to recapitulate human B cell receptor repertoires comparable to those found in PBMCs^123,124^. However, a significant limitation is the lack of a fully supportive microenvironment for the maturation of human HSC-derived B cells^121^. B cell maturation is a complex process requiring cooperative interactions among innate immune cell, stromal cell and T_FH_ cell. In these complicated process, IL-6 and IL-7 are critical cytokines to regulate B cell maturation and facilitate the formation of lymphoid follicles^125,126^. In addition, the constitutive expression of human IL-3, GM-CSF, and SCF transgenes for human myeloid cell development indirectly supports B cell maturation. This is attributed to IL-4 produced by these innate effector cells, which drives B cells to spontaneously produce polyclonal human antibodies^127,128^. Following DENV-2 (dengue virus serotype-2) infection in NSG-SGM3 HIS mice, an antigen-specific IgG response was detected^128^. These findings highlight the essential role of human cytokines in enabling HIS mice to investigate human B cell development and immune responses.

In addition to cytokine signaling, HLA expression has a critical role to improve human T cell development for T–B cell cooperation. Lentiviral vector-mediated overexpression of human HLA-DR4, GM-CSF, and IFN-α in HIS mice spleen and liver enhanced B cell and CD4^+^ T cell maturation. Following immunization with human cytomegalovirus gB antigen, these mice demonstrated detectable antigen-specific IgG and IgA, confirming the occurrence of class-switching^129^. Thus, the orchestrated delivery of HLA and cytokines has the potential to improve human B cell maturation in HIS mice.

#### The secondary/tertiary lymphoid organs and antigen presentation

Lymphoid organs provide specialized microenvironments that regulate the development and activation of lymphocytes. The absence or incomplete development of draining lymph nodes in HIS mice results in immune responses predominantly localized to spleen and liver^130–132^. However, the incomplete lymph node development in HIS mice limits preclinical research by disrupting key immune processes, including antigen presentation, lymphocyte activation, cytokine signaling, and memory formation. TSLP is a critical mediator of lymphoid organ reconstitution and lymphocyte development, exhibiting structural and functional homology to IL-7 and signaling through CD127 (refs.^133,134^). Backcrossing TSLP transgenic mice onto the BRGS strain to generate BRGST HIS mice significantly enhanced both lymph node formation and lymphocyte development. Furthermore, increased IgG and IgA levels in the BRGST HIS mice HIV disease model indicate improved class-switch recombination and humoral immune responses^53^. The transplantation of human lymphoid organoids generated from pluripotent stem cells provides an alternative way to provide functional immune response microenvironments. Bouffi et al.^135^ transplanted human intestinal organoids under the kidney capsule of HIS mice. These organoids developed follicle-like structures that showed colocalization of human T and B cells, along with the presence of plasma cells and IgA expression, indicating robust B cell development and maturation.

### Exogenous estrogen

Estrogen, a critical female sex hormone, has a pivotal role in the development and maintenance of the female reproductive system and the expression of secondary sexual characteristics. The four major endogenous estrogens, estrone (E1), estradiol (E2), estriol (E3), and estetrol (E4), are synthesized in specific tissues: E1 and E2 are primarily ovarian products, whereas E3 and E4 are predominantly synthesized during pregnancy, by the placenta and fetal liver, respectively^136,137^.

**Fig. 2:**
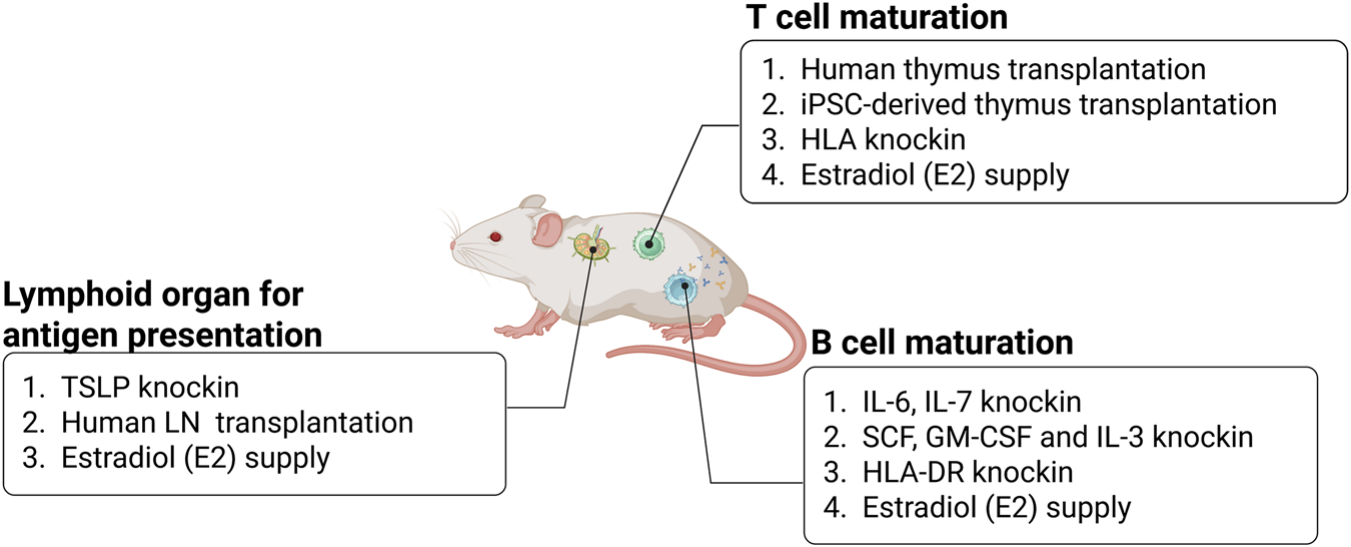
Summary of improved human adaptive immunity in HIS mice. Transplanting human tissue is the most ideal way to support the human adaptive immune system development in human immune system (HIS) mice. However, ethical concerns and unstable resource availability prevent this method from being commonly used for preclinical testing by all researchers. Alternative approaches to support human adaptive immune development include the overexpression of human cytokines/chemokines or HLA in HIS mice. Recently, studies have demonstrated that estradiol treatment enhances the maturation of T cells, B cells, and lymphoid organs, offering a simpler method for establishing HIS mice. GM-CSF granulocyte–macrophage colony-stimulating factor, iPSC induced pluripotent stem cell, SCF stem cell factor, TSLP thymic stromal lymphopoietin, LN lymph node.

E2 mediates immune system development and function through interactions with ERα and ERβ, which are expressed in hematopoietic cells and a broad range of immune cells, including B cells, T cells, dendritic cells, and macrophages. However, the immunoregulatory roles of E1, E3, and E4 remain to be explored^138–140^. Supplementing NBSGW-HIS mice (THX mice) with E2 has been shown to improve immune cell development and function. This supplementation induced human TEC differentiation in mice thymus, which is crucial for human HSC-derived T cell maturation. THX mice treated with antigen demonstrated T cell-dependent and T cell-independent antigen-specific B cell responses characterized by class-switching and somatic hypermutation. Analysis of ligand–receptor interactions for V–J segment recombination revealed similarities between humans and THX mice. Both T cell and B cell clonality expanded and diversified. In addition, the generation of high-affinity, neutralizing antibodies was detected in S*.*Typhi flagellin immunization and SARS-CoV-2 vaccination models. A “butterfly rash” (malar rash) and kidney glomerular IgG deposition were observed in SLE^95^. These findings demonstrated the THX mice model’s broad applicability for studying protective immunity in infectious diseases and autoimmune pathogenesis (Fig. 2).

### Human TME

The TME is a complex system shaped by both tumor cells and infiltrating immune cells. The immune cells including T_reg_ cells, M2-polarized macrophages, and MDSCs in TME support tumor growth via immune suppression^141,142^. These cells establish an immunosuppressive niche by modulating cytokine networks, inhibiting antigen presentation, and remodeling the extracellular matrix. However, HIS mice exhibit limitations in the development of these critical immune populations owing to species-specific cytokine incompatibilities and restricted hematopoietic lineage differentiation^143,144^. To overcome these challenges, several mouse strains, such as NSG-Flt3 and MISTRG mice, have been engineered to express human cytokines (for example, Flt3L, M-CSF, IL-3, and GM-CSF). This promotes the development of M2 macrophages and MDSCs, thereby improving the recapitulation of the TME for preclinical research^32^.

HLA mismatch introduces a significant complexity in HIS mice tumor models. The expression of matched HLA is crucial for proper T cell education. However, HLA incompatibility between the engrafted tumor and the host immune system can substantially impede tumor growth and progression^144^. Furthermore, even with successful immune engraftment, mismatched HLA can elicit alloreactive responses, leading to tumor rejection or impaired growth. This challenge is particularly found in huPBMC HIS mice model; mature T cells attack HLA-mismatched tumor grafts^143,145^. To address these limitations, strategies such as HLA matching via genome editing or autologous tumor-immune co-engraftment have been explored. These systems effectively avoid alloreactivity and enhance tumor engraftment^146^.

## Preclinical HIS mice platform bridging to clinical translation

**Fig. 3:**
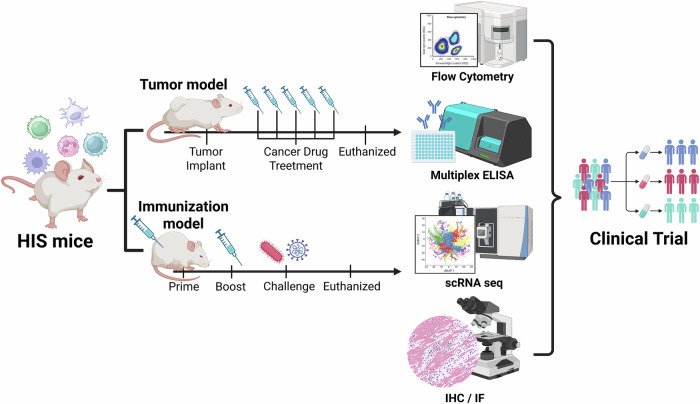
Workflow for the preclinical evaluation of cancer and immunization using a HIS mice platform. Human immune system (HIS) mice provide various models with both cellular and humoral immune responses for preclinical evaluation. The human data derived from these models are more compelling and informative compared with those from traditional mouse models. By using single-cell RNA-sequencing (scRNA-seq) analysis, researchers can identify the cellular targets affected by drug treatment within the HIS mouse platform. These mechanistic and safety data are highly valuable for informing clinical trials. ELISA enzyme-linked immunosorbent assay, IF immunofluorescence, IHC immunohistochemistry.

HIS mice models offer a unique advantage by allowing researchers to investigate human immune responses in vivo, representing a significant progression in preclinical cancer and vaccination research (Fig. 3). In light of increasing research costs, ethical considerations, and challenges with experimental reproducibility, these models have become preferred platforms for exploring immunotherapeutic approaches. Since 2020, a growing number of therapies have demonstrated therapeutic potential in HIS mice models before preclinical testing, reinforcing their relevance in translating laboratory findings into clinical applications^147^ (Table 5).

**Table 5:** HIS mice for preclinical application.

Treatment strategy	Cancer model	HIS mice	Treatment	Clinical trial	Ref.
Combination therapy	Myeloproliferative neoplasms	CD34^+^ hematopoietic stem cell (HSC) human immune system (HIS)	Ruxolitinib and JQ1	NCT02158858	^148^
	Non-small-cell lung cancer	CD34^+^ HSC HIS	MitoQ and cisplatin	–	^149^
	Non-small-cell lung cancer	Human peripheral blood mononuclear cell (huPBMC) HIS	Pembrolizumab and bevacizumab	NCT02366143	^150^
	Colorectal cancer	CD34^+^ HSC HIS	Cabozantinib and nivolumab	NCT04963283	^79^
	Inflammatory breast cancer	CD34^+^ HSC HIS	Panitumumab and anti-PD-L1 Ab	NCT05177796	^151^
Cell-based immunotherapy	Acute myeloid leukemia	CD34^+^ HSC HIS	Chimeric antigen receptor (CAR)-T cells, checkpoint inhibitors, and bispecific antibodies	–	^152^
Novel drug therapy	Breast cancer	CD34^+^ HSC HIS	CRISPR–Cas9-conjugated FCPCV nanoparticles to CCL5	–	^153^
	Colorectal adenocarcinoma	huPBMC HIS	Tri-specific nano-antibody (Tri-Nab)	–	^154^

### Combination therapy

Combination therapy has become a fundamental approach in cancer treatment. HIS tumor models have been instrumental in evaluating the effectiveness of such strategies. In a myelofibrosis (MF) PDX HIS mice model, the combination of ruxolitinib and the BET inhibitor JQ1 demonstrated therapeutic efficacy by inhibiting the JAK–STAT pathway in myeloproliferative neoplasms (ClinicalTrials.gov Identifier: NCT02158858)^148^.

In studies of solid tumors, the combination of cisplatin and the mitochondria-targeted antioxidant MitoQ has demonstrated efficacy in an NSCLC HIS mice model. This combined treatment disrupted the ROS/HIF-1α/LAP signaling cascade, enhancing cisplatin-induced Vγ9δ2 T-lymphocyte cytotoxicity^149^. Beyond chemotherapy-antioxidant synergy, combining anti-PD-1 (pembrolizumab) and bevacizumab (antivascular endothelial growth factor) inhibited tumor growth and prevented postoperative recurrence in an NSCLC HIS mice model by increasing CD8^+^ granzyme B^+^ T cell infiltration (ClinicalTrials.gov Identifier: NCT02366143)^150^. Reflecting clinical outcomes, cabozantinib plus nivolumab (Ca/N) elicited stronger immune activity and higher TIL infiltration than cobimetinib plus atezolizumab (Co/A) in a CRC HIS mice model. These findings mirror the clinical failure of the Co/A regimen (ClinicalTrials.gov Identifier: NCT04963283)^79^. Furthermore, the epidermal growth factor receptor-targeted drug panitumumab combined with anti-PD-L1 antibody therapy potently suppressed inflammatory breast cancer in HIS mice model (ClinicalTrials.gov Identifier: NCT05177796)^151^.

### Cell-based immunotherapy

In addition to combination approaches, cell-based immunotherapies have become promising strategies for targeting cancer with high specificity. HIS models have played a crucial role in evaluating these therapies within clinically relevant environments. Xu et al.^152^ applied a multipronged approach involving checkpoint inhibitors (pembrolizumab, atezolizumab, and ipilimumab), CAR-T cells, and bispecific antibody (blinatumomab) in acute myeloid leukemia, illustrating the therapeutic differences and potential synergies among these methods.

### Novel drug development

HIS tumor models have also contributed significantly to the development of novel target for cancer therapy. Yan et al.^153^ utilized nanoparticles to deliver CRISPR–Cas9, specifically targeting and disrupting the chemokine CCL5, a key contributor to tumor-associated immunosuppression and metastasis. The disruption of CCL5 pathway significantly enhanced immune-mediated breast tumor suppression by remodeling the TME in a HIS mouse model. Ye et al.^154^ developed tri-specific nano-antibodies by immobilizing antibodies against PD-L1, 4-1BB, and NKG2A onto biocompatible albumin/polyester composite nanoparticles. This innovative design facilitates the activation and proliferation of NK cells and CD8^+^ T cells at tumor sites. Enhancing the interaction between NK cells and CD8^+^ T cells with cancer cells promotes antitumor immunity in a colorectal adenocarcinoma HIS mice model.

### Challenges and prospects for standardization

Despite the promising applications of HIS mice in preclinical research, a critical challenge to their broader adoption lies in the lack of standardization across laboratories. Variability in mouse strains, human cell sources (for example, cord blood, peripheral blood, and fetal liver), and HIS establishment protocols can significantly influence the extent of immune humanization. These discrepancies ultimately compromise experimental reproducibility and translational comparability.

To address these challenges, several strategies have been proposed. The establishment of shared human HSC banks with well-characterized donors would help reduce interlaboratory variability. In parallel, standardized engraftment protocols would further improve experimental consistency. Notably, the MISHUM (Minimal Information for Standardization of Humanized Mice) framework was proposed by Stripecke et al.^155^ as an international initiative to standardize reporting on mouse strains, HSC donor traits, engraftment methods, and immune engraftment outcomes. Together, these efforts aim to improve the reproducibility and transparency of HIS mice studies.

In addition, mice model resource centers that provide HIS mice offer a stable and reliable source of models, which can facilitate more reproducible drug evaluation. Furthermore, prospective comparisons between HIS mouse outcomes and patient responses would help define the predictive value of these models. Such validation efforts would strengthen the utility of HIS mice in drug development pipelines and enhance their acceptance in regulatory evaluation.

## Conclusion

HIS mice are a powerful tool in translational biomedical research, offering notable advantages for clinical applications. By engrafting HIS components, these models allow for more reliable evaluations of drug efficacy, toxicity, and immune responses. Over the past decade, continuous progress in HIS mice models, particularly in tumor and infection studies, has proven valuable for predicting clinical outcomes and informing medical indications. These models are crucial for novel drug development and combination therapy testing. The rise of ICIs has further broadened the utility of HIS mice in nanodrug development and combination treatments. This broad applicability emphasizes their essential role in preclinical evaluations of diverse therapeutic strategies, including combination treatments, cell-based immunotherapies, and next-generation drugs.

Nevertheless, the broader ethical implications of HIS mice models warrant careful consideration. Beyond thymic transplantation concerns, issues related to animal welfare and the use of human-derived materials remain important considerations for the field. Complementary approaches, including organoid co-cultures and organ-on-a-chip platforms, may provide valuable alternatives in selected experimental contexts, in alignment with the “Replace, Reduce, Refine” (3R) principles and reinforcing commitments to responsible and humane research practices.

Despite these considerations, HIS mice remain indispensable for translational in vivo biomedical research of human immune responses. As novel therapeutic approaches continue to arise, these models will remain indispensable for translating research into impactful medical applications.
